# Multi-detector CT imaging: impact of virtual tube current reduction and sparse sampling on detection of vertebral fractures

**DOI:** 10.1007/s00330-019-06090-2

**Published:** 2019-03-22

**Authors:** Nico Sollmann, Kai Mei, Dennis M. Hedderich, Christian Maegerlein, Felix K. Kopp, Maximilian T. Löffler, Claus Zimmer, Ernst J. Rummeny, Jan S. Kirschke, Thomas Baum, Peter B. Noël

**Affiliations:** 1 Department of Diagnostic and Interventional Neuroradiology, Klinikum rechts der Isar, Technische Universität München, Ismaninger Str. 22, 81675 Munich, Germany; 2 TUM-Neuroimaging Center, Klinikum rechts der Isar, Technische Universität München, Munich, Germany; 3 Department of Diagnostic and Interventional Radiology, Klinikum rechts der Isar, Technische Universität München, Ismaninger Str. 22, 81675 Munich, Germany; 4 Department of Radiology, Perelman School of Medicine, University of Pennsylvania, 3400 Spruce Street, One Silverstein, Philadelphia, PA 19104 USA

**Keywords:** Bone fractures, Multi-detector computed tomography, Osteoporosis, Radiation exposure, Spine

## Abstract

**Purpose:**

To systematically evaluate the effects of virtual tube current reduction and sparse sampling on image quality and vertebral fracture diagnostics in multi-detector computed tomography (MDCT).

**Materials and methods:**

In routine MDCT scans of 35 patients (80.0% females, 70.6 ± 14.2 years, 65.7% showing vertebral fractures), reduced radiation doses were retrospectively simulated by virtually lowering tube currents and applying sparse sampling, considering 50%, 25%, and 10% of the original tube current and projections, respectively. Two readers evaluated items of image quality and presence of vertebral fractures. Readout between the evaluations in the original images and those with virtually lowered tube currents or sparse sampling were compared.

**Results:**

A significant difference was revealed between the evaluations of image quality between MDCT with virtually lowered tube current and sparse-sampled MDCT (*p* < 0.001). Sparse-sampled data with only 25% of original projections still showed good to very good overall image quality and contrast of vertebrae as well as minimal artifacts. There were no missed fractures in sparse-sampled MDCT with 50% reduction of projections, and clinically acceptable determination of fracture age was possible in MDCT with 75% reduction of projections, in contrast to MDCT with 50% or 75% virtual tube current reduction, respectively.

**Conclusion:**

Sparse-sampled MDCT provides adequate image quality and diagnostic accuracy for vertebral fracture detection with 50% of original projections in contrast to corresponding MDCT with lowered tube current. Thus, sparse sampling is a promising technique for dose reductions in MDCT that could be introduced in future generations of scanners.

**Key Points:**

*• MDCT with a reduction of projection numbers of 50% still showed high diagnostic accuracy without any missed vertebral fractures.*

*• Clinically acceptable determination of vertebral fracture age was possible in MDCT with a reduction of projection numbers of 75%.*

*• With sparse sampling, higher reductions in radiation exposure can be achieved without compromised image or diagnostic quality in routine MDCT of the spine as compared to MDCT with reduced tube currents.*

**Electronic supplementary material:**

The online version of this article (10.1007/s00330-019-06090-2) contains supplementary material, which is available to authorized users.

## Introduction

Vertebral fractures are frequent in clinical routine and are primarily observed in the context of injuries or as major manifestations of osteoporosis, even in the absence of any obvious trauma [1–3]. Spine radiography is commonly applied for the detection of suspected vertebral fractures; however, it has been shown that computed tomography (CT) is superior by reducing the risk of missing a fracture, thus resulting in a higher sensitivity and specificity with fracture detection rates of 97 to 100% at the spine [4–6].

The increased use of CT instead of radiography for the purpose of improved diagnostics comes at the cost of elevated radiation exposure for the patient: one-time scanning with a modern CT scanner applies an estimated effective dose of 5.6 mSv and 10.0 mSv for the lumbar and whole dorsal spine, respectively [7, 8]. The use of CT entails an estimated cancer risk ratio that is multifold higher than in radiography, and it can further increase due to cumulative effects when additional imaging is performed [7–9]. Thus, CT with reduced radiation exposure, but without simultaneous constraints for image quality or diagnostic accuracy seems crucial.

Despite its clinical relevance, previous research on dose reductions in CT at the spine is generally scarce. In vivo, radiation exposure reductions have been achieved by lowered tube current or voltage at the level of the cervical spine, resulting in largely preserved image quality except for the lower cervical spine [10, 11]. Recently, iterative reconstruction (IR) algorithms have been applied together with low-dose CT, but led to worse image quality for soft tissue and cervical vertebrae when compared to standard-dose CT using filtered back projection (FBP) [12]. To date, only few studies investigated CT with reduced doses specifically for diagnostics of vertebral fractures, showing that low-dose CT with IR may maintain a high diagnostic performance compared to standard-dose CT with IR in trauma patients [13, 14].

In addition to lowering tube current or voltage to reduce x-ray exposure during CT, the number of acquired projections can be decreased with sparse-sampled acquisition schemes. Reducing projection views is a promising strategy since lowering the number of projections can clearly reduce the radiation dose, with previous research indicating a high potential of this approach resulting in reasonable image quality [15–20]. However, sparse sampling has not been applied at the spine for fracture diagnostics yet.

Against this background, the aim of this study is to evaluate the effects of virtual tube current reduction and sparse sampling on image quality and vertebral fracture diagnostics in multi-detector CT (MDCT). Our hypothesis is that MDCT with sparse sampling would provide better image and diagnostic quality when compared to MDCT with virtual lowering of tube current and, thus, might allow for more drastic reductions in radiation exposure.

## Materials and methods

### Patients

This retrospective study was approved by the local institutional review board (registration number: 62/18S) and was conducted in accordance with the Declaration of Helsinki. Overall, 35 patients were included (80.0% females, mean age: 70.6 ± 14.2 years, age range: 26.2–93.1 years), with 23 patients (65.7%) showing at least one vertebral fracture (fracture group) and 12 patients (34.3%) showing no vertebral fracture (control group). Eligible patient cases were identified in the institutional digital Picture Archiving and Communication System (PACS) between November 2016 and November 2017.

Inclusion criteria were (1) availability of routine MDCT of the spine (irrespective of the initial suspected diagnosis or distinct clinical symptoms leading to MDCT), (2) additional spinal magnetic resonance imaging (MRI) performed prior or subsequent to MDCT (including short-tau inversion recovery sequences; only for the fracture group), and (3) diagnosis of at least one vertebral fracture according to MDCT (only for the fracture group). Exclusion criteria for both the fracture and control group were (1) age below 18 years, (2) movement artifacts in imaging data, (3) malignant bone lesions (e.g., bone metastases), (4) any history of metabolic bone disorders aside from osteoporosis, and (5) any implants captured by the field of view (FOV).

### Multi-detector computed tomography

All scans were performed with a 64-channel MDCT scanner (Brilliance 64; Philips Healthcare). Parameters of the scanning protocol are shown in Table 1. X-ray tube current was modulated implicitly by the scanner during the helical scan according to the examined body part and estimated body size as derived from the scout scan. All examinations were performed without administration of a contrast agent.

**Table 1:** Scan parameters and image reconstruction

Scan parameters	
Rotation time	1 s (62.9% of subjects), 0.75 s (37.1% of subjects)
Pitch	0.608 (62.9% of subjects), 0.953 (37.1% of subjects)
Tube voltage	120 kV
Tube current	143.4 ± 76.0 mA (49.2–326.6 mA)
Exposure	180.4 ± 87.7 mAs (68.0–459.0 mAs)
Volumetric CT dose index	11.7 ± 5.7 mGy (4.4–29.7 mGy)
Image reconstruction	
Field of view	200 mm × 200 mm
Slice thickness	0.30 mm
Voxel spacing	0.39 × 0.39 × 0.30 mm^3^
Voxel resolution	Restricted with respect to the fixed collimator width of the detector pixel (0.625 mm)
Reformations	Sagittal, axial, coronal
Windowing	Individually adjustable. Standard settings: bone window (window width, 2500 HU; window center, 500 HU) and soft-tissue window (window width, 360 HU; window center, 60 HU)

### Virtual tube current reduction and sparse sampling

Based on raw projection data, we used a simulation algorithm to generate lower tube currents for MDCT scans [16, 21–23]. System parameters of the scanner were considered and electronic noise was calibrated for each pixel at the detector. Simulations were generated as if the scans were made at 50% (D50 P100), 25% (D25 P100), and 10% (D10 P100) of the original x-ray tube current and used for image evaluation, in addition to the original imaging data defined as D100 P100. Furthermore, sparse sampling was applied at levels of 50% (D100 P50), 25% (D100 P25), and 10% (D100 P10) of the original projection data, which was achieved by reading every second, fourth, and tenth projection angle and deleting the remaining projections in the sinogram [16, 23, 24]. While the projection number per full rotation was lowered, other parameters, including patient location and projection geometry, were not changed. All images were reconstructed using FBP and a standard Ram-Lak filter [25, 26]. Table 1 provides an overview of image reconstruction parameters.

### Image evaluation

Two board-certified radiologists (6 and 8 years of experience in radiology) evaluated all imaging data (35 patients × 7 imaging datasets per patient = 245 datasets for evaluation for reader 1 [R1] and reader 2 [R2], respectively), which were uploaded and stored in IntelliSpace Portal (version 9.0; Philips Healthcare) for visualization and evaluation.

First, both readers evaluated the original images with 100% tube current and projections (D100 P100) as the clinical standard in consensus with the MRI available to confirm the diagnosis of an acute or old vertebral fracture. Then, both readers independently evaluated the remaining datasets in random order (D50 P100, D25 P100, D10 P100, D100 P50, D100 P25, and D100 P10), assessing images derived from the same tube current or number of projections within 1 day for all patients and sticking to an interval of at least 7 days before continuing with images of another tube current or number of projections. The order of patient cases was also randomized for each tube current and number of projections, with the readers being blinded to all clinical patient data, the evaluations of each other, the assignments of patients to the fracture or control group, and all previous evaluations performed.

During evaluations, the number of vertebral fractures per patient had to be determined first, with the vertebrae included in the FOV being provided for each case to allow assignment of single fractures to specific vertebrae. Then, the items and scores presented in Table 2 were considered.

**Table 2:** Scoring system for the evaluation of image quality and vertebral fractures

Overall image evaluation					
Item	Score				
	1	2	3	4	5
Overall image quality	Very good to perfect quality	Good to very good quality	Medium quality	Poor quality	Vertebrae not distinguishable
	No compromise of diagnostic quality	No compromise of diagnostic quality	Acceptable diagnostic quality	Unacceptable diagnostic quality	
Overall artifacts	No artifacts	Minimal artifacts	Prominent artifacts	Major artifacts	Vertebrae not distinguishable
	No compromise of diagnostic quality	No compromise of diagnostic quality	Acceptable diagnostic quality	Unacceptable diagnostic quality	
Contrast of vertebrae	Very good to perfect contrast	Good to very good contrast	Medium contrast	Poor contrast	Vertebrae not distinguishable
	No compromise of diagnostic quality	No compromise of diagnostic quality	Acceptable diagnostic quality	Unacceptable diagnostic quality	
Fracture evaluation					
Item	Score				
	1	2		3	
Diagnostic confidence	High confidence	Medium confidence		Low confidence	
Age of fracture	Acute	Unclear/not distinguishable		Old	

### Statistical analyses

For statistical analyses and generation of graphs, SPSS (version 20.0; IBM SPSS Statistics for Windows, IBM Corp.) was used. A *p* value < 0.05 was considered statistically significant.

Descriptive statistics were calculated for patient demographics and all items of evaluation. Wilcoxon signed-rank tests were performed to compare overall image quality, overall artifacts, contrast of vertebrae, and diagnostic confidence between MDCT with virtually lowered tube current and sparse-sampled images, i.e., D50 P100 vs. D100 P50, D25 P100 vs. D100 P25, and D10 P100 vs. D100 P10, respectively. Furthermore, overall image quality, overall artifacts, and contrast of vertebrae of D100 P100 as the gold standard were compared with MDCT with virtually lowered tube current and sparse-sampled images using Wilcoxon signed-rank tests, which were achieved separately for each reader.

Interreader intraclass correlation coefficients (ICCs) were calculated for overall image quality, overall artifacts, contrast of vertebrae, and diagnostic confidence in MDCT with virtually lowered tube current and sparse sampling, respectively [27, 28]. As a measure of agreement between imaging with virtual lowering of tube current and sparse sampling, Cohen’s kappa coefficients were determined for age of fracture. Moreover, weighted Cohen's kappa coefficients were determined between the results of both readers for age of fracture [29–31].

## Results

**Fig. 1:**
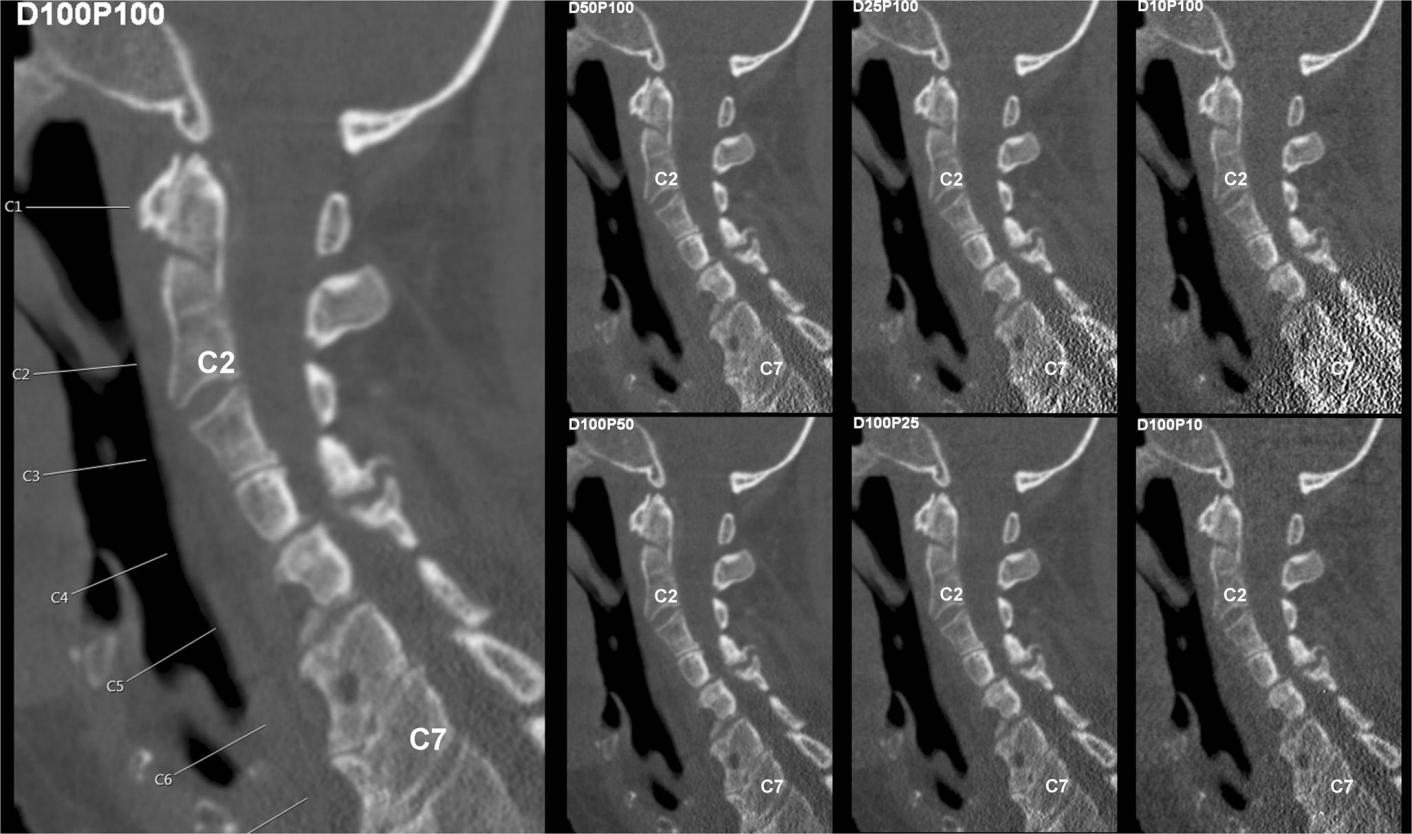
Virtual tube current reduction and sparse sampling in multi-detector CT (MDCT) of the cervical spine. Sagittal slices derived from full-dose MDCT (D100 P100), MDCT with virtually lowered tube current (D50 P100, D25 P100, and D10 P100), and MDCT with sparse sampling (D100 P50, D100 P25, and D100 P10) are shown in a patient with a cervical fracture (C2, dens fracture)

**Fig. 2:**
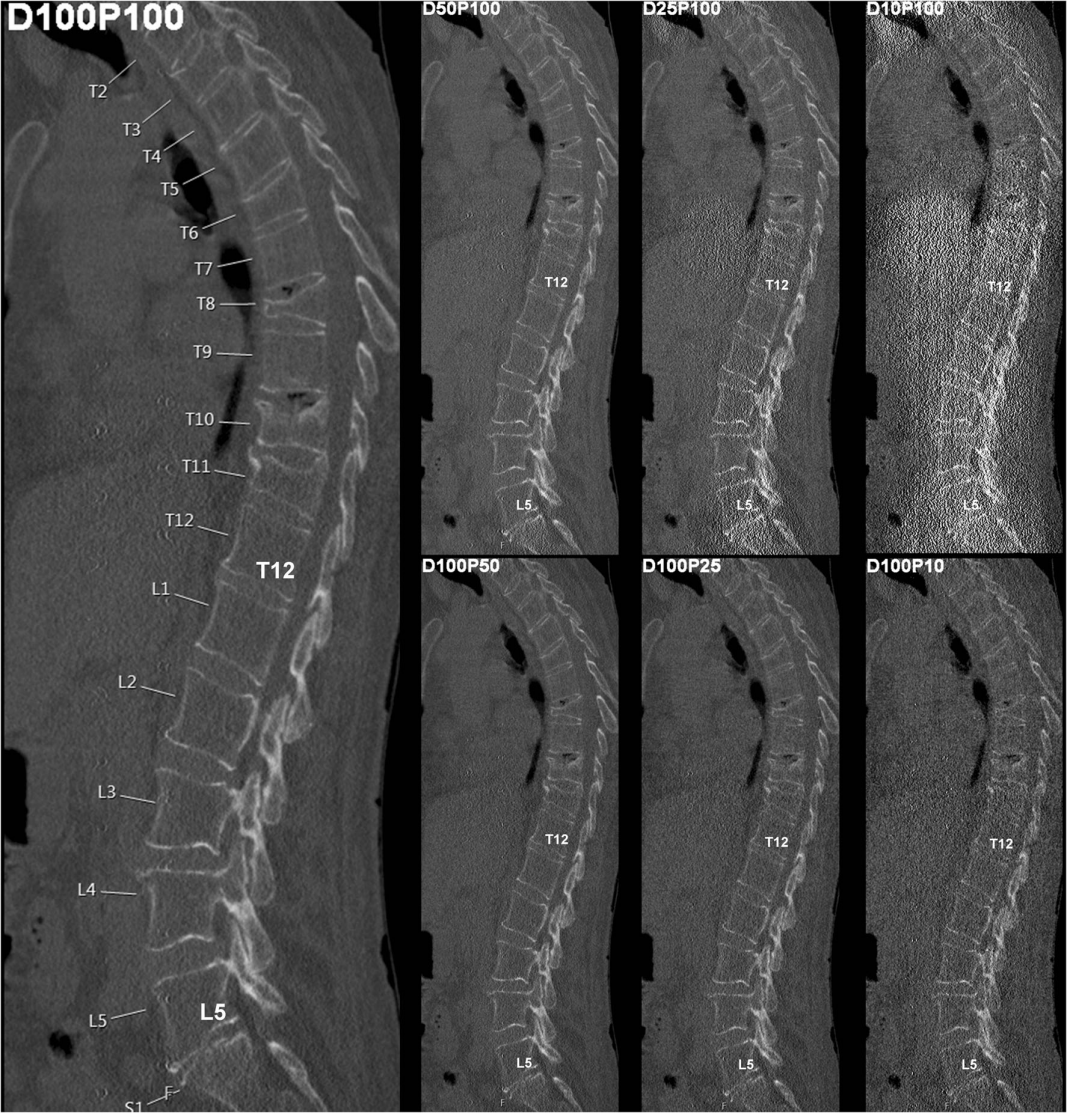
Virtual tube current reduction and sparse sampling in multi-detector CT (MDCT) of the thoracic and lumbar spine. Sagittal slices derived from full-dose MDCT (D100 P100), MDCT with virtually lowered tube current (D50 P100, D25 P100, and D10 P100), and MDCT with sparse sampling (D100 P50, D100 P25, and D100 P10) are shown in a patient with five thoracic fractures (T3, T6, T8, T10, and T11)

Virtual lowering of tube current and sparse sampling were successfully achieved in all patients (Figs. 1 and 2). A median of eight vertebrae (range 4–19 vertebrae) was captured by the FOV of MDCT scans, which covered the cervical spine in 20.0%, the cervico-thoracic spine in 8.6%, the thoracic spine in 5.7%, the thoraco-lumbar spine in 28.6%, and the lumbar spine in 37.1%. The average volumetric CT dose index recorded in the dose reports was 11.7 ± 5.7 mGy for original MDCT scans (Table 1), and was amounted 5.9 mGy, 2.9 mGy, and 1.2 mGy for MDCT with virtually lowered tube current or sparse sampling at 50%, 25%, and 10% of original data, respectively.

Both readers correctly identified all patients of the control group (34.3% of included patients) without any assignments of vertebral fractures to controls in MDCT with virtually lowered tube current or sparse sampling. Among patients of the fracture group (65.7% of included patients), a total of 48 vertebral fractures was observed in the original MDCT with 100% tube current and projections (D100 P100). Patients of the fracture group showed a median of two vertebral fractures (range 1–6 vertebral fractures). These fractures affected the cervical spine in 10.0%, the thoracic spine in 40.0%, and the lumbar spine in 50.0%. Based on original MDCT and MRI scanning, vertebral fractures were diagnosed as acute in 58.3% and old in 41.7%.

### Overall image evaluation

Both virtual tube current reduction and sparse sampling led to decreased overall image quality, increased overall artifacts, and reduced contrast of vertebrae according to the evaluation of both readers (Table 3, Figs. 1 and 2, Supplementary Fig. 1). The assessed parameters were significantly different in MDCT with virtually lowered tube current and sparse-sampled datasets as compared to those in D100 P100 (*p* < 0.001; D100 P100 vs. D50 P100/D25 P100/D10 P100 and D100 P100 vs. D100 P50/D100 P25/D100 P10 of both readers).

**Table 3:** Overall image evaluation

Overall Image Quality										
	D100 P100	D50 P100	D100 P50	*p*-value	D25 P100	D100 P25	*p*-value	D10 P100	D100 P10	*p*-value
R1	*1.03 ± 0.17*	*1.74 ± 0.56*	*1.31 ± 0.47*	<0.001	*2.80 ± 0.68*	*2.17 ± 0.45*	<0.001	*3.91 ± 0.51*	*3.23 ± 0.43*	<0.001
R2	*1.03 ± 0.17*	*1.77 ± 0.55*	*1.31 ± 0.47*	<0.001	*2.89 ± 0.58*	*2.20 ± 0.47*	<0.001	*3.97 ± 0.57*	*3.26 ± 0.44*	<0.001
Interreader ICC	-	0.98	0.93	-	0.94	0.97	-	0.89	0.96	-
Overall Artifacts										
	D100 P100	D50 P100	D100 P50	*p*-value	D25 P100	D100 P25	*p*-value	D10 P100	D100 P10	*p*-value
R1	*1.17 ± 0.38*	*1.86 ± 0.69*	*1.57 ± 0.56*	<0.001	*2.71 ± 0.67*	*2.17 ± 0.38*	<0.001	*4.03 ± 0.71*	*2.97 ± 0.51*	<0.001
R2	*1.17 ± 0.38*	*1.86 ± 0.65*	*1.60 ± 0.60*	0.20	*2.86 ± 0.65*	*2.20 ± 0.47*	<0.001	*4.14 ± 0.69*	*3.14 ± 0.65*	<0.001
Interreader ICC	-	0.93	0.88	-	0.91	0.87	-	0.94	0.81	-
Contrast of Vertebrae										
	D100 P100	D50 P100	D100 P50	*p*-value	D25 P100	D100 P25	*p*-value	D10 P100	D100 P10	*p*-value
R1	*1.17 ± 0.38*	*1.69 ± 0.53*	*1.17 ± 0.38*	<0.001	*2.63 ± 0.69*	*2.11 ± 0.58*	<0.001	*3.77 ± 0.77*	*2.91 ± 0.56*	<0.001
R2	*1.17 ± 0.38*	*1.71 ± 0.57*	*1.26 ± 0.44*	<0.001	*2.66 ± 0.77*	*2.29 ± 0.67*	0.005	*3.89 ± 0.68*	*3.06 ± 0.59*	<0.001
Interreader ICC	-	0.92	0.86	-	0.96	0.88	-	0.94	0.88	-

Results of overall image evaluation as mean ± standard deviation for overall image quality, overall artifacts, and contrast of vertebrae according to the evaluation of reader 1 (R1) and reader 2 (R2). Results are separately provided for multi-detector CT (MDCT) with virtually lowered tube current (D50 P100, D25 P100, and D10 P100) and sparse-sampled MDCT (D100 P50, D100 P25, and D100 P10) as well as original imaging (D100 P100). Interreader intraclass correlation coefficients (ICCs) and *p* values are shown for the comparison of MDCT with virtually lowered tube current against sparse-sampled MDCT

**Fig. 3:**
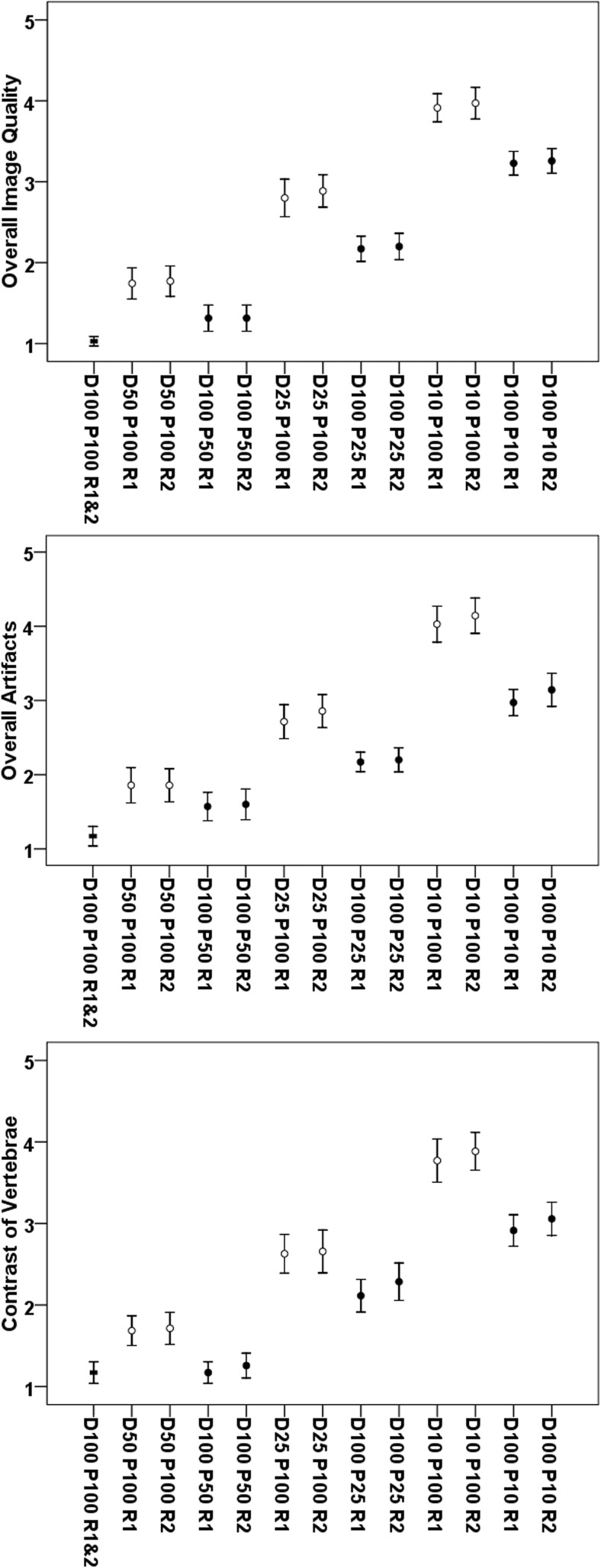
Overall image evaluation. This figure depicts the mean scores ± standard deviation for overall image quality, overall artifacts, and contrast of vertebrae according to the evaluation of reader 1 (R1) and reader 2 (R2). Blank circles show results for multi-detector CT (MDCT) with virtually lowered tube current (D50 P100, D25 P100, and D10 P100), whereas black circles visualize the results derived from sparse-sampled MDCT (D100 P50, D100 P25, and D100 P10). The black square represents the results for original MDCT with 100% of tube current and 100% of projections (D100 P100)

When comparing MDCT with virtually lowered tube current to sparse-sampled datasets for overall image quality, sparse sampling resulted in significantly better scores according to each reader for all comparisons (*p* < 0.001, D50 P100 vs. D100 P50, D25 P100 vs. D100 P25, and D10 P100 vs. D100 P10 of both readers; Table 3 and Fig. 3). Similar findings with better scores for sparse-sampled imaging than for MDCT with virtually lowered tube current were observed for overall artifacts (*p* < 0.001, except for D50 P100 vs. D100 P50 for R2: *p* = 0.20; Table 3 and Fig. 3) and contrast of vertebrae (*p* < 0.001, except for D25 P100 vs. D100 P25 for R2: *p* = 0.005; Table 3 and Fig. 3). Good interreader agreement was observed for overall image quality, overall artifacts, and contrast of vertebrae, respectively (ICC > 0.80, R1 vs. R2 for D50 P100/D25 P100/D10 P100 and D100 P50/D100 P25/D100 P10; Table 3).

### Fracture evaluation

Virtual tube current reduction by 50% of original current (D50 P100) allowed for correct detection of 100% (R1) and 95.8% (R2) of vertebral fractures when compared to original MDCT. Further lowering to 10% of original current (D10 P100) resulted in correct detection of 79.2% (R1) and 87.5% (R2) of vertebral fractures (Table 4). Sparse-sampled MDCT with 50% of the original projections (D100 P50) allowed for correct detection of all vertebral fractures by both readers as compared to original MDCT. Further decreasing the number of projections down to 10% of the original data allowed for correct detection of 95.8% (R1) and 91.7% (R2) of vertebral fractures (Table 4).

**Table 4:** Fracture evaluation

Reported Number of Fractures										
	D100 P100	D50 P100	D100 P50	-	D25 P100	D100 P25	-	D10 P100	D100 P10	-
R1	*48*	*48*	*48*		*47*	*47*		*38*	*46*	
R2	*48*	*46*	*48*		*45*	*45*		*42*	*44*	
Diagnostic Confidence										
	D100 P100	D50 P100	D100 P50	*p*-value	D25 P100	D100 P25	*p*-value	D10 P100	D100 P10	*p*-value
R1	*1.02 ± 0.14*	*1.17 ± 0.38*	*1.13 ± 0.39*	0.48	*1.83 ± 0.79*	*1.34 ± 0.48*	<0.001	*2.55 ± 0.69*	*1.87 ± 0.58*	<0.001
R2	*1.02 ± 0.14*	*1.15 ± 0.36*	*1.10 ± 0.31*	0.41	*1.80 ± 0.79*	*1.29 ± 0.46*	<0.001	*2.45 ± 0.71*	*1.80 ± 0.51*	<0.001
Interreader ICC	-	0.91	0.96	-	0.98	0.91	-	0.97	0.98	-
Age of Fracture (acute / unclear / old)										
	D100 P100	D50 P100	D100 P50	Kappa	D25 P100	D100 P25	Kappa	D10 P100	D100 P10	Kappa
R1	*28/0/20*	*27/2/19*	*27/1/20*	0.84	*18/20/9*	*26/2/19*	0.42	*4/30/4*	*16/24/6*	0.24
R2	*28/0/20*	*27/3/16*	*26/1/21*	0.79	*16/22/7*	*25/3/17*	0.35	*4/34/4*	*15/24/5*	0.13
Interreader Weighted Kappa	-	0.96	0.92	-	0.93	0.96	-	0.89	0.92	-

Results regarding the reported number of fractures, diagnostic confidence (mean ± standard deviation), and age of fracture (absolute numbers for acute/unclear/old vertebral fractures) according to the evaluation of multi-detector CT (MDCT) by reader 1 (R1) and reader 2 (R2). Results are separately provided for virtually lowered tube current (D50 P100, D25 P100, and D10 P100), sparse-sampled MDCT (D100 P50, D100 P25, and D100 P10), and the original dose (D100 P100). Interreader intraclass correlation coefficients (ICCs) and *p* values derived from the comparison of MDCT with virtually lowered tube current against sparse-sampled MDCT are provided for diagnostic confidence. Cohen’s kappa coefficients are depicted for agreement of reported age of detected vertebral fractures between MDCT with virtually lowered tube current and sparse-sampled MDCT, and weighted Cohen’s kappa is shown for interreader evaluation

**Fig. 4:**
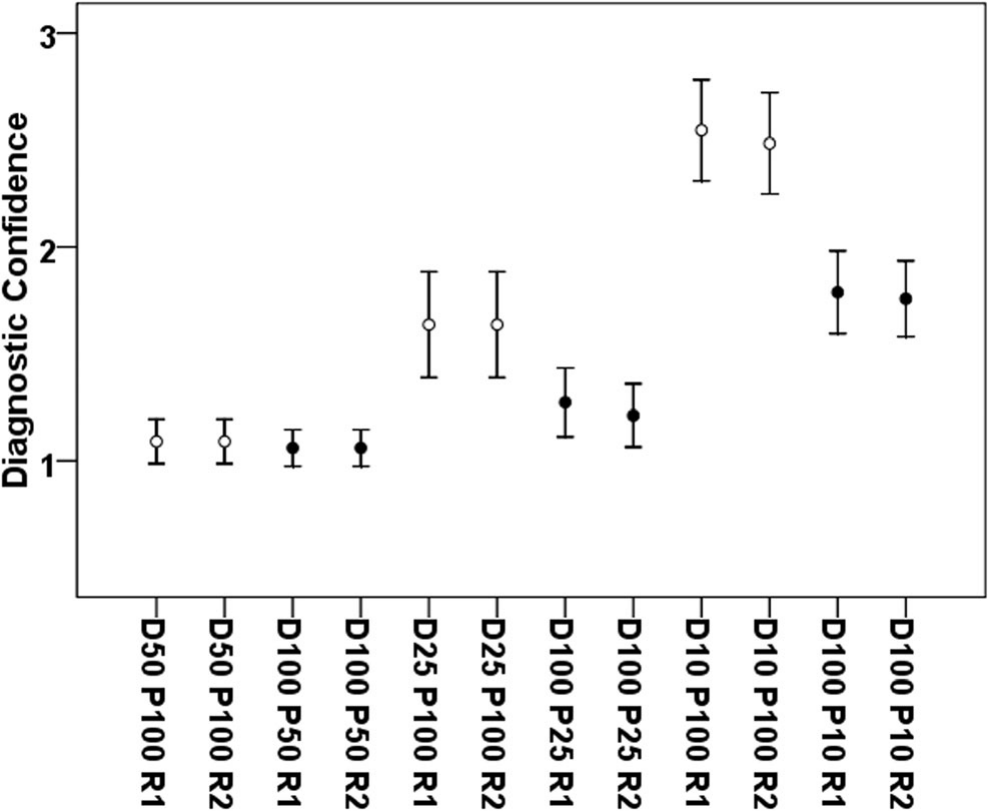
Diagnostic confidence. This figure depicts the mean scores ± standard deviation for diagnostic confidence according to the evaluation of reader 1 (R1) and reader 2 (R2). Blank circles show results for multi-detector CT (MDCT) with virtually lowered tube current (D50 P100, D25 P100, and D10 P100), whereas black circles visualize the results derived from sparse-sampled MDCT (D100 P50, D100 P25, and D100 P10)

Both readers reported preserved high diagnostic confidence for both virtual lowering of tube current and lowered projection numbers down to 50% of original MDCT without a significant difference in scores (*p* = 0.48 for R1 and *p* = 0.41 for R2; Table 4 and Fig. 4). For MDCT with 25% or 10% of original projections, average diagnostic confidence was still high (D100 P25) to medium (D100 P10), and it was medium (D25 P100) to low (D10 P100) when MDCT with virtually lowered tube current was considered (Table 4 and Fig. 4). Correspondingly, a significant difference was observed between MDCT with virtually lowered tube current and sparse-sampled imaging at 25% or 10% of original tube current or projections (*p* < 0.001 for both readers; Table 4 and Fig. 4). Excellent agreement between the evaluations of both readers was observed for both virtual tube current reductions and sparse sampling down to 10% of projections of original imaging data (ICC > 0.90, R1 vs. R2 for D50 P100/D25 P100/D10 P100 and D100 P50/D100 P25/D100 P10; Table 4).

Concerning the age of reported vertebral fractures, sparse sampling showed better results regarding the differentiation between acute, old, and unclear fracture age (Table 4). For sparse-sampled MDCT at 25% of original projections, fracture age was determined as unclear in 4.3% (R1) and 6.7% (R2) of detected vertebral fractures. According to imaging with 25% of original tube current, 42.6% (R1) and 48.9% (R2) of detected vertebral fractures were of unclear age (Table 4). Excellent agreement was observed in the evaluations of fracture age between readers (kappa > 0.88; Table 4).

## Discussion

This study investigated the effects of virtual tube current reduction and sparse sampling on image quality and diagnostic accuracy of vertebral fractures in MDCT. When comparing virtual tube current reductions to sparse sampling, superior results for image quality and fracture diagnostics were evident for sparse-sampled MDCT. Specifically, no missed vertebral fractures occurred for MDCT with a reduction of 50% in projection numbers, and determination of fracture age was still reliably possible in MDCT with a reduction in projection numbers of 75%.

CT is increasingly applied for first-line diagnostics of vertebral fractures due to its high sensitivity and specificity and excellent fracture detection rates [4–6]. However, clearly higher estimated effective doses of 5.6 mSv for the lumbar and 10.0 mSv for the whole dorsal spine in CT compared with radiography result in a considerably increased risk of developing cancer later in life [7–9]. Importantly, cancer risks are summative, and radiography or CT performed for initial diagnostics are not the only sources of radiation exposure, with a patient suffering from an acute traumatic vertebral fracture being exposed to a cumulative effective dose of about 38 mSv only during inpatient stay and without taking into account later follow-up imaging [32]. Consequently, reduction of CT-related radiation exposure is necessary, but should ideally be achieved without loss of image quality or diagnostic accuracy. Despite evident clinical relevance, only a limited body of literature distinctly evaluated approaches for radiation exposure reductions in CT of the spine.

Concerning CT with reduced doses, a small increase in image noise and no difference in subjective image quality evaluation was reported for cervical structures, allowing dose reductions of 61–71% [11]. Low-kV CT with reduced radiation doses by approximately 34% demonstrated good image quality for structures of the neck, but compromised image quality for the lower cervical spine [10]. In patients with lumbar disc herniation, simulated low-dose CT with reductions in tube charge settings to 65% of the standard dose were considered adequate for diagnostic purposes previously [33]. Furthermore, IR algorithms have been introduced for CT with reduced doses, leading to better image quality for intervertebral discs, neural foramina, and ligaments, but worse image quality for vertebrae when compared with standard-dose CT using FBP [12]. Ultra-low-dose CT may still provide an acceptable image quality and exhibited a diagnostic accuracy similar to that of low-dose CT in patients with chronic lumbar back pain [34].

To the authors’ knowledge, only few recent studies investigated CT with reduced doses specifically for diagnostics of vertebral fractures. The diagnostic performance of lumbar low-dose CT (47–69% radiation dose reduction) combined with IR was comparable to that of standard-dose CT with IR [13]. Higher levels of IR for low-dose CT (50% radiation dose reduction) still provided high image quality and diagnostic confidence [14]. In contrast to these studies, we simulated tube current reduction, which allows for intra-subject comparisons between standard- and low-dose MDCT with virtually lowered tube currents down to even 10% of original imaging. Thus, low-dose MDCT was simulated with relative dose reduction steps in comparison to original MDCT, which enables systematic virtual tube current reductions as a fraction of the initially performed, optimal scanning protocol according to the scanner’s automatic tube current modulation.

We further applied sparse sampling, which is novel for diagnostics of vertebral fractures. So far, assessments of bone mineral density and microstructure at the spine derived from MDCT with sparse sampling have been performed, with sparse-sampled imaging appearing more robust in comparison to MDCT with virtually lowered tube currents [16, 17]. In the present study, sparse sampling was superior in terms of overall image quality, overall artifacts, and contrast of vertebrae when compared with MDCT with virtually lowered tube current (Table 3 and Fig. 3). These results were obtained with good to excellent correlations between two experienced readers for MDCT with virtually lowered tube currents and sparse sampling, respectively (Table 3 and Fig. 3). Furthermore, sparse sampling led to superior results in detecting vertebral fractures, with no missed fractures for D100 P50 in contrast to D50 P100 (Table 4). Diagnostic confidence and correct determination of fracture age was better for sparse sampling, with clinically acceptable determination of fracture age in D100 P25 compared with D25 P100 (Table 4 and Fig. 4).

There are limitations to this study. First, it is not yet possible to apply sparse sampling at commercial MDCT scanners, thus restricting direct clinical applicability. However, first results from a prototype were recently reported, indicating that sparse sampling for MDCT could become broadly available in future generations of MDCT scanners [19, 20]. Second, we used FBP instead of IR, but IR has the potential to provide increased image quality particularly for imaging with reduced doses [35–37]. The use of algorithms taking advantage of artificial intelligence for image reconstructions might further regularly improve image quality in the near future [37]. Third, we solely enrolled patients with vertebral fractures and without implants, such as spinal instrumentation. Thus, upcoming studies may evaluate sparse sampling in cohorts with spinal implants to distinctly evaluate whether sparse sampling is also beneficial and even superior to tube current restrictions when implant-related metal artifacts are present in MDCT. Fourth, the retrospective design and the comparatively small patient cohort have to be acknowledged as a limitation. Prospective approaches including more patients are needed to confirm the results of the present study.

In conclusion, our results demonstrate the feasibility of using sparse sampling for fracture detection at the spine, with clear superiority as compared to MDCT with virtual reduction of tube current. Therefore, sparse sampling represents a promising option that might allow for even more drastic radiation dose reductions while revealing better image quality and diagnostic characteristics than MDCT with tube current reduction does.

## Electronic supplementary material

ESM 1 (DOCX 3036 kb)

## Abbreviations

CT: Computed tomography
FBP: Filtered back projection
FOV: Field of view
ICC: Intraclass correlation coefficient
IR: Iterative reconstruction
MDCT: Multi-detector computed tomography
MRI: Magnetic resonance imaging
PACS: Picture Archiving and Communication System
R1: Reader 1
R2: Reader 2
