# Supplementary material for: Multi-detector CT imaging: impact of virtual tube current reduction and sparse sampling on detection of vertebral fractures
Source: Eur Radiol. 2019 Mar 22;29(7):3606–16. doi: 10.1007/s00330-019-06090-2 (PMC6554251; doi:10.1007/s00330-019-06090-2)
Supplement: Supplementary file 1 — (DOCX 3036 kb) [file 330_2019_6090_MOESM1_ESM.docx]

**SUPPLEMENTARY MATERIAL**


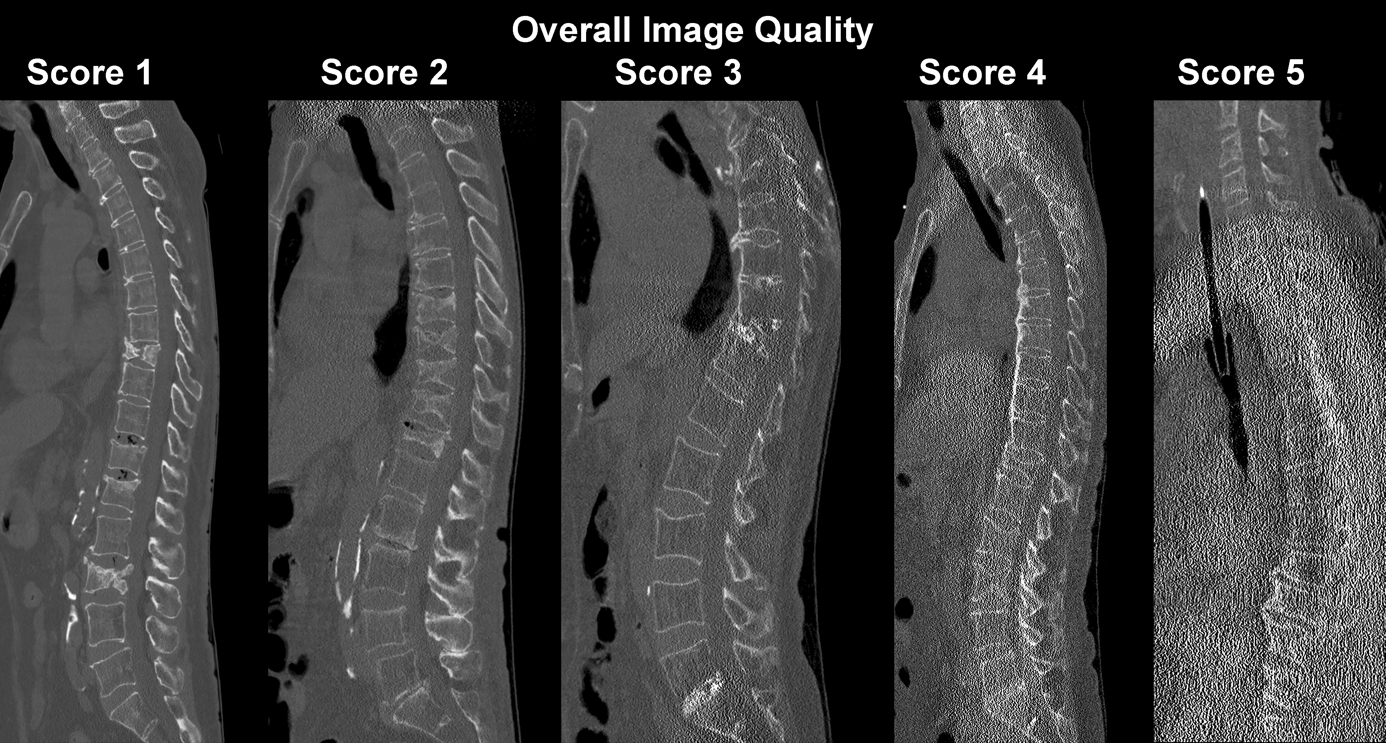


**Supplementary Figure 1:** Exemplary patient cases regarding evaluation of overall image quality
